# Diaqua­(5-carb­oxy­benzene-1,3-di­carboxyl­ato-κ*O* ^1^)(4,4′-dimethyl-2,2′-bipyridine-κ^2^ *N*,*N*′)zinc

**DOI:** 10.1107/S1600536811019519

**Published:** 2011-05-28

**Authors:** Chong-Zhen Mei, Kai-Hui Li, Wen-Wen Shan

**Affiliations:** a Institute of Environmental and Municipal Engineering, North China University of Water Conservancy and Electric Power, Zhengzhou 450011, People’s Republic of China

## Abstract

In the title compound, [Zn(C_9_H_4_O_6_)(C_12_H_12_N_2_)(H_2_O)_2_], the Zn^II^ atom is five-coordinated by two N atoms from a 4,4′-dimethyl-2,2′-bipyridine ligand, one O atom from a 5-carb­oxy­benzene-1,3-dicarboxyl­ate ligand and two water mol­ecules in a distorted trigonal–bipyramidal geometry. The complex mol­ecules are linked by inter­molecular O—H⋯O hydrogen bonds and partly overlapping π–π inter­actions [centroid–centroid distance = 4.017 (2) Å] into a three-dimensional supra­molecular network.

## Related literature

For background to the network topologies and applications of coordination polymers, see: Maspoch *et al.* (2007 ▶); Ockwig *et al.* (2005 ▶); Zang *et al.* (2006 ▶). For O—H⋯O hydrogen bonds, see: Desiraju *et al.* (2004 ▶). For π–π inter­actions, see: Zang *et al.* (2010 ▶).
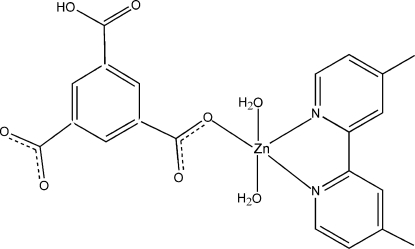


## Experimental

### 

#### Crystal data

[Zn(C_9_H_4_O_6_)(C_12_H_12_N_2_)(H_2_O)_2_]
*M* *_r_* = 493.76
Triclinic, 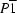

*a* = 9.1938 (9) Å
*b* = 10.7978 (8) Å
*c* = 11.5842 (7) Å
α = 85.238 (6)°
β = 72.960 (7)°
γ = 69.760 (8)°
*V* = 1031.40 (16) Å^3^
*Z* = 2
Mo *K*α radiation
μ = 1.24 mm^−1^
*T* = 296 K
0.21 × 0.20 × 0.19 mm

#### Data collection

Bruker APEXII CCD diffractometer
Absorption correction: multi-scan (*SADABS*; Bruker, 2001 ▶) *T*_min_ = 0.780, *T*_max_ = 0.798
7701 measured reflections
3607 independent reflections
3246 reflections with *I* > 2σ(*I*)
*R*_int_ = 0.032

#### Refinement

*R*[*F* ^2^ > 2σ(*F* ^2^)] = 0.032
*wR*(*F* ^2^) = 0.089
*S* = 1.05
3607 reflections
292 parameters
H-atom parameters constrained
Δρ_max_ = 0.34 e Å^−3^
Δρ_min_ = −0.28 e Å^−3^

### 

Data collection: *APEX2* (Bruker, 2007 ▶); cell refinement: *SAINT* (Bruker, 2007 ▶); data reduction: *SAINT*; program(s) used to solve structure: *SHELXS97* (Sheldrick, 2008 ▶); program(s) used to refine structure: *SHELXL97* (Sheldrick, 2008 ▶); molecular graphics: *DIAMOND* (Brandenburg, 1999 ▶); software used to prepare material for publication: *SHELXTL* (Sheldrick, 2008 ▶).

## Supplementary Material

Crystal structure: contains datablocks I, global. DOI: 10.1107/S1600536811019519/hy2433sup1.cif

Structure factors: contains datablocks I. DOI: 10.1107/S1600536811019519/hy2433Isup2.hkl

Additional supplementary materials:  crystallographic information; 3D view; checkCIF report

## Figures and Tables

**Table 1:** Hydrogen-bond geometry (Å, °)

*D*—H⋯*A*	*D*—H	H⋯*A*	*D*⋯*A*	*D*—H⋯*A*
O5—H5*A*⋯O6^i^	0.82	1.79	2.603 (2)	171
O1*W*—H1*WA*⋯O1^ii^	0.84	1.81	2.635 (2)	165
O1*W*—H1*WB*⋯O2^iii^	0.84	1.75	2.593 (2)	171
O2*W*—H2*WA*⋯O2^iv^	0.85	1.86	2.688 (2)	166
O2*W*—H2*WB*⋯O4^v^	0.84	1.79	2.633 (2)	176

Symmetry codes: (i) 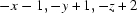; (ii) 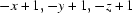; (iii) 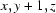; (iv) 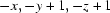; (v) 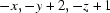.

**Fig. 1.**
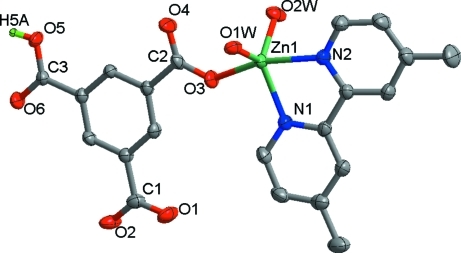
Molecular structure of the title compound. Displacement ellipsoids are drawn at the 50% probability level. Irrespective H atoms are omitted for clarity.

**Fig. 2.**
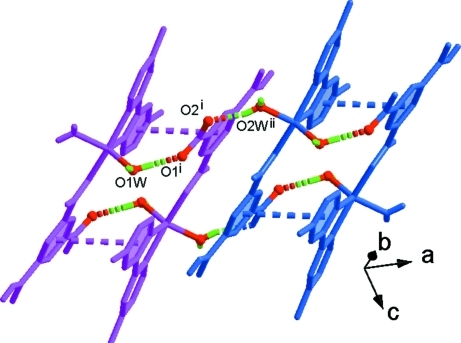
A view of the supramolecular chain in the title compound. Dashed lines represent hydrogen bonds and π–π interactions. [Symmetry codes: (i) 1-x, 1-y, 1-z; (ii) 1+x, y, z.]

**Fig. 3.**
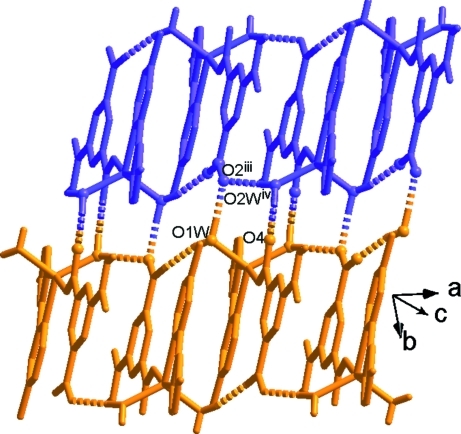
A view of the two-dimensional supramolecular structure in the title compound. Dashed lines represent hydrogen bonds. [Symmetry codes: (iii) x, 1+y, z; (iv) -x, 2-y, 1-z.]

**Fig. 4.**
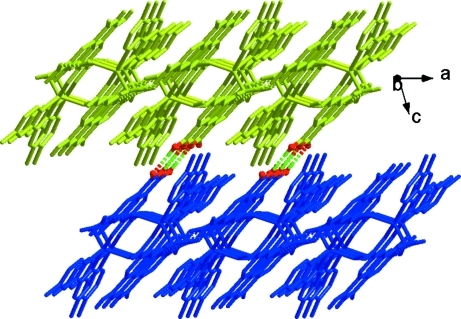
The three-dimensional supramolecular structure connected by interlayer hydrogen bonds (dashed lines).

## supplementary crystallographic information

### Comment

Supramolecular coordination assemblies have received much attention not only for their variety of architectures but also for the potential applications as functional materials (Maspoch *et al.*, 2007; Ockwig *et al.*, 2005). A great number of multidentate organic ligands such as organic aromatic polycarboxylate ligands and *N*-donor building blocks have been successfully employed in the generation of many interesting systems (Zang *et al.*, 2006). To further explore the influence of multicarboxylate and *N*-donor ligands on the properties and construction of coordination compounds, we undertake the synthetic and structural studies on a Zn(II) complex based on benzene-1,3,5-tricarboxylic acid (H_3_btc) and 4,4'-dimethyl-2,2'-bipyridine (dmbpy), Zn(Hbtc)(bmbpy)(H_2_O)_2_.

As shown in Fig. 1, the asymmetric unit consists of one Zn^II^ atom, one Hbtc ligand, one dmbpy ligand and two coordinated water molecules. The Hbtc ligand occurs in a form with an intact COOH group. The metal ion is coordinated by one O atom from the Hbtc ligand, two O atoms of water molecules and two N atoms from the chelating dmbpy ligand, completing a distorted trigonal bipyramidal geometry. N1, O1W and O2W comprise the equatorial plane, while O3 and N2 occupy the axial positions. A pair of symmetry-related complex molecules are associated together through O1W—H1WA···O1^i^ hydrogen bonds (Table 1) [symmetry code: (i) 1-*x*, 1-*y*, 1-*z*], forming a dimeric unit, in which π–π stacking interaction occurs with a centroid–centroid distance of 4.017 (2)Å between two face-to-face aromatic rings (phenyl ring and pyridine ring bearing the N1 atom). Adjacent dimeric units are connected by O2W—H2WA···O2^ii^ hydrogen bonds [symmetry code: (ii) -*x*, 1-*y*, 1-*z*], resulting in a one-dimensional supramolecular chain running along the *a*-axis (Fig. 2). As depicted in Fig. 3, the chains are extended to a two-dimensional supramolecular structure through O1W—H1WB···O2^iii^ and O2W—H2WB···O4^iv^ hydrogen bonds [symmetry codes: (iii) *x*, 1+*y*, *z*; (iv) -*x*, 2-*y*, 1-*z*]. The hydroxyl group and the uncoordinated O atom of the intact COOH group serve as donor and accepter, respectively. Neighboring such carboxylic groups are linked together through O5—H5A···O6^v^ hydrogen bonds [symmetry code: (v) -1-*x*, 1-*y*, 2-*z*]. Thus, the layers are interconnected into a three-dimensional supramolecular structure (Fig. 4).

### Experimental

The title compound was synthesized hydrothermally in a Teflon-lined stainless steel container by heating a mixture of benzene-1,3,5-tricarboxylic acid (0.011 g, 0.05 mmol), 4,4'-dimethyl-2,2'-bipyridine (0.009 g, 0.05 mmol), Zn(NO_3_)_2_.6H_2_O (0.015 g, 0.05 mmol) and NaOH (0.004 g, 0.1 mmol) in 7 ml of distilled water at 120°C for 3 d, and then cooled to room temperature. Colorless block crystals of the title compound were obtained in 68% yield based on zinc.

### Refinement

H atoms on C atoms and hydroxyl O atom were positioned geometrically and refined as riding atoms, with C—H = 0.93 (aromatic) and 0.96 (methyl) and O—H = 0.82 Å and with *U*_iso_(H) = 1.2(1.5 for methyl and hydroxyl)*U*_eq_(C,O). Water H atoms were obtained from a difference Fourier map and restrained to ideal configuration of the water molecules and fixed in the final stages of refinement, with O—H = 0.85 Å and *U*_iso_(H) = 1.5*U*_eq_(O).

### Crystal data

**Table:**

[Zn(C_9_H_4_O_6_)(C_12_H_12_N_2_)(H_2_O)_2_]	*Z* = 2
*M**_r_* = 493.76	*F*(000) = 508
Triclinic, *P*1	*D*_x_ = 1.590 Mg m^−^^3^
Hall symbol: -P 1	Mo *K*α radiation, λ = 0.71073 Å
*a* = 9.1938 (9) Å	Cell parameters from 4795 reflections
*b* = 10.7978 (8) Å	θ = 3.5–29.1°
*c* = 11.5842 (7) Å	µ = 1.24 mm^−^^1^
α = 85.238 (6)°	*T* = 296 K
β = 72.960 (7)°	Block, colorless
γ = 69.760 (8)°	0.21 × 0.20 × 0.19 mm
*V* = 1031.40 (16) Å^3^	

### Data collection

**Table:**

Bruker APEXII CCD diffractometer	3607 independent reflections
Radiation source: fine-focus sealed tube	3246 reflections with *I* > 2σ(*I*)
graphite	*R*_int_ = 0.032
φ and ω scans	θ_max_ = 25.0°, θ_min_ = 3.5°
Absorption correction: multi-scan (*SADABS*; Bruker, 2001)	*h* = −10→10
*T*_min_ = 0.780, *T*_max_ = 0.798	*k* = −12→12
7701 measured reflections	*l* = −13→13

### Refinement

**Table:**

Refinement on *F*^2^	Primary atom site location: structure-invariant direct methods
Least-squares matrix: full	Secondary atom site location: difference Fourier map
*R*[*F*^2^ > 2σ(*F*^2^)] = 0.032	Hydrogen site location: inferred from neighbouring sites
*wR*(*F*^2^) = 0.089	H-atom parameters constrained
*S* = 1.05	*w* = 1/[σ^2^(*F*_o_^2^) + (0.0516*P*)^2^] where *P* = (*F*_o_^2^ + 2*F*_c_^2^)/3
3607 reflections	(Δ/σ)_max_ = 0.009
292 parameters	Δρ_max_ = 0.34 e Å^−^^3^
0 restraints	Δρ_min_ = −0.28 e Å^−^^3^

### Fractional atomic coordinates and isotropic or equivalent isotropic displacement parameters (Å^2^)

**Table:**

	*x*	*y*	*z*	*U*_iso_*/*U*_eq_	
Zn1	0.29959 (3)	0.80971 (2)	0.41463 (2)	0.02705 (12)	
O1	0.3344 (2)	0.21247 (17)	0.51057 (18)	0.0451 (5)	
O2	0.1654 (2)	0.11417 (16)	0.62226 (18)	0.0417 (5)	
O3	0.1815 (2)	0.68960 (16)	0.50550 (15)	0.0353 (4)	
O4	−0.0341 (2)	0.81358 (16)	0.64551 (17)	0.0442 (5)	
O5	−0.4023 (2)	0.60517 (15)	0.91517 (15)	0.0378 (4)	
H5A	−0.4810	0.5991	0.9684	0.057*	
O6	−0.3417 (2)	0.38720 (15)	0.90916 (15)	0.0377 (4)	
O1W	0.34722 (19)	0.87467 (15)	0.55069 (14)	0.0310 (4)	
H1WA	0.4474	0.8605	0.5349	0.046*	
H1WB	0.2920	0.9551	0.5668	0.046*	
O2W	0.1124 (2)	0.92769 (15)	0.35858 (16)	0.0380 (4)	
H2WA	0.0254	0.9106	0.3765	0.057*	
H2WB	0.0912	1.0101	0.3581	0.057*	
N1	0.4831 (2)	0.64334 (18)	0.31684 (17)	0.0294 (4)	
N2	0.4584 (2)	0.89292 (18)	0.27789 (18)	0.0313 (5)	
C1	0.2061 (3)	0.2140 (2)	0.5853 (2)	0.0295 (5)	
C2	0.0560 (3)	0.7072 (2)	0.5941 (2)	0.0277 (5)	
C3	−0.3103 (3)	0.4905 (2)	0.8723 (2)	0.0257 (5)	
C4	0.0878 (3)	0.3468 (2)	0.6388 (2)	0.0244 (5)	
C5	−0.0533 (3)	0.3572 (2)	0.7292 (2)	0.0261 (5)	
H5	−0.0770	0.2816	0.7590	0.031*	
C6	−0.1601 (3)	0.4811 (2)	0.7756 (2)	0.0254 (5)	
C7	−0.1244 (3)	0.5936 (2)	0.73168 (19)	0.0246 (5)	
H7	−0.1955	0.6759	0.7635	0.030*	
C8	0.0167 (3)	0.5847 (2)	0.6404 (2)	0.0241 (5)	
C9	0.1210 (3)	0.4604 (2)	0.5950 (2)	0.0256 (5)	
H9	0.2154	0.4532	0.5337	0.031*	
C10	0.4961 (3)	0.5176 (2)	0.3444 (2)	0.0352 (6)	
H10	0.4184	0.5014	0.4101	0.042*	
C11	0.6183 (3)	0.4113 (2)	0.2805 (2)	0.0378 (6)	
H11	0.6214	0.3259	0.3027	0.045*	
C12	0.7357 (3)	0.4315 (2)	0.1840 (2)	0.0341 (6)	
C13	0.8735 (4)	0.3183 (3)	0.1126 (3)	0.0495 (7)	
H13A	0.9377	0.2685	0.1638	0.074*	
H13B	0.9394	0.3519	0.0463	0.074*	
H13C	0.8313	0.2621	0.0821	0.074*	
C14	0.7234 (3)	0.5617 (2)	0.1547 (2)	0.0319 (6)	
H14	0.8002	0.5794	0.0894	0.038*	
C15	0.5972 (3)	0.6652 (2)	0.2222 (2)	0.0269 (5)	
C16	0.5789 (3)	0.8062 (2)	0.1965 (2)	0.0278 (5)	
C17	0.6761 (3)	0.8476 (2)	0.0971 (2)	0.0374 (6)	
H17	0.7560	0.7855	0.0409	0.045*	
C18	0.6557 (3)	0.9805 (2)	0.0801 (2)	0.0384 (6)	
C19	0.7596 (4)	1.0268 (3)	−0.0278 (3)	0.0604 (9)	
H19A	0.8531	1.0303	−0.0091	0.091*	
H19B	0.6990	1.1132	−0.0489	0.091*	
H19C	0.7930	0.9666	−0.0946	0.091*	
C20	0.5346 (3)	1.0673 (3)	0.1677 (3)	0.0436 (7)	
H20	0.5178	1.1574	0.1616	0.052*	
C21	0.4393 (3)	1.0211 (2)	0.2634 (2)	0.0398 (6)	
H21	0.3581	1.0815	0.3204	0.048*	

### Atomic displacement parameters (Å^2^)

**Table:**

	*U*^11^	*U*^22^	*U*^33^	*U*^12^	*U*^13^	*U*^23^
Zn1	0.02375 (18)	0.02181 (17)	0.03013 (18)	−0.00935 (12)	0.00191 (12)	0.00227 (11)
O1	0.0236 (10)	0.0374 (10)	0.0601 (12)	−0.0075 (8)	0.0085 (9)	−0.0093 (9)
O2	0.0267 (9)	0.0214 (8)	0.0687 (13)	−0.0072 (7)	−0.0004 (9)	−0.0071 (8)
O3	0.0319 (10)	0.0301 (9)	0.0400 (10)	−0.0185 (7)	0.0036 (8)	0.0062 (7)
O4	0.0418 (11)	0.0204 (8)	0.0559 (12)	−0.0106 (8)	0.0066 (9)	0.0036 (8)
O5	0.0326 (10)	0.0273 (9)	0.0371 (10)	−0.0088 (8)	0.0132 (8)	−0.0019 (7)
O6	0.0350 (10)	0.0280 (9)	0.0419 (10)	−0.0160 (8)	0.0064 (8)	0.0056 (8)
O1W	0.0220 (9)	0.0236 (8)	0.0419 (10)	−0.0063 (7)	−0.0016 (8)	−0.0038 (7)
O2W	0.0299 (10)	0.0224 (8)	0.0614 (12)	−0.0118 (7)	−0.0109 (9)	0.0087 (8)
N1	0.0298 (11)	0.0249 (10)	0.0308 (10)	−0.0114 (8)	−0.0025 (9)	0.0018 (8)
N2	0.0269 (11)	0.0269 (10)	0.0334 (11)	−0.0088 (9)	0.0008 (9)	0.0014 (9)
C1	0.0227 (13)	0.0257 (12)	0.0387 (14)	−0.0066 (10)	−0.0073 (11)	−0.0044 (10)
C2	0.0272 (13)	0.0243 (12)	0.0321 (13)	−0.0128 (10)	−0.0062 (11)	0.0086 (10)
C3	0.0262 (12)	0.0240 (12)	0.0236 (11)	−0.0099 (10)	−0.0011 (10)	0.0021 (9)
C4	0.0211 (12)	0.0230 (11)	0.0294 (12)	−0.0099 (9)	−0.0048 (10)	0.0009 (9)
C5	0.0281 (13)	0.0202 (11)	0.0304 (12)	−0.0121 (10)	−0.0052 (10)	0.0046 (9)
C6	0.0237 (12)	0.0256 (11)	0.0258 (11)	−0.0107 (10)	−0.0033 (10)	0.0029 (9)
C7	0.0232 (12)	0.0196 (11)	0.0270 (12)	−0.0075 (9)	−0.0008 (10)	0.0003 (9)
C8	0.0220 (12)	0.0240 (11)	0.0256 (11)	−0.0099 (9)	−0.0040 (10)	0.0037 (9)
C9	0.0198 (12)	0.0273 (12)	0.0286 (12)	−0.0122 (9)	−0.0004 (10)	0.0016 (10)
C10	0.0410 (15)	0.0290 (13)	0.0341 (13)	−0.0167 (11)	−0.0040 (12)	0.0064 (11)
C11	0.0471 (17)	0.0233 (12)	0.0422 (15)	−0.0103 (11)	−0.0149 (13)	0.0060 (11)
C12	0.0369 (15)	0.0291 (13)	0.0359 (14)	−0.0065 (11)	−0.0144 (12)	−0.0011 (11)
C13	0.0533 (19)	0.0326 (14)	0.0516 (17)	−0.0004 (13)	−0.0134 (15)	−0.0062 (13)
C14	0.0288 (14)	0.0317 (13)	0.0305 (13)	−0.0088 (10)	−0.0033 (11)	0.0012 (10)
C15	0.0244 (12)	0.0296 (12)	0.0249 (12)	−0.0096 (10)	−0.0043 (10)	0.0030 (10)
C16	0.0250 (13)	0.0258 (12)	0.0303 (12)	−0.0090 (10)	−0.0050 (10)	0.0033 (10)
C17	0.0323 (14)	0.0343 (13)	0.0343 (14)	−0.0098 (11)	0.0054 (12)	0.0006 (11)
C18	0.0371 (15)	0.0327 (13)	0.0425 (15)	−0.0160 (12)	−0.0046 (12)	0.0101 (12)
C19	0.063 (2)	0.0489 (18)	0.062 (2)	−0.0310 (16)	0.0061 (17)	0.0128 (16)
C20	0.0425 (16)	0.0268 (13)	0.0559 (17)	−0.0132 (12)	−0.0060 (14)	0.0082 (12)
C21	0.0383 (15)	0.0256 (12)	0.0456 (15)	−0.0087 (11)	0.0008 (13)	−0.0008 (11)

### Geometric parameters (Å, °)

**Table:**

Zn1—O1W	1.9922 (16)	C7—C8	1.392 (3)
Zn1—O2W	2.0018 (16)	C7—H7	0.9300
Zn1—O3	2.0115 (16)	C8—C9	1.389 (3)
Zn1—N1	2.1159 (19)	C9—H9	0.9300
Zn1—N2	2.1826 (19)	C10—C11	1.374 (4)
O1—C1	1.238 (3)	C10—H10	0.9300
O2—C1	1.262 (3)	C11—C12	1.370 (4)
O3—C2	1.268 (3)	C11—H11	0.9300
O4—C2	1.235 (3)	C12—C14	1.391 (3)
O5—C3	1.275 (3)	C12—C13	1.506 (4)
O5—H5A	0.8200	C13—H13A	0.9600
O6—C3	1.257 (3)	C13—H13B	0.9600
O1W—H1WA	0.8444	C13—H13C	0.9600
O1W—H1WB	0.8446	C14—C15	1.387 (3)
O2W—H2WA	0.8460	C14—H14	0.9300
O2W—H2WB	0.8421	C15—C16	1.487 (3)
N1—C10	1.341 (3)	C16—C17	1.382 (3)
N1—C15	1.346 (3)	C17—C18	1.386 (3)
N2—C21	1.336 (3)	C17—H17	0.9300
N2—C16	1.346 (3)	C18—C20	1.386 (4)
C1—C4	1.516 (3)	C18—C19	1.501 (4)
C2—C8	1.505 (3)	C19—H19A	0.9600
C3—C6	1.480 (3)	C19—H19B	0.9600
C4—C5	1.384 (3)	C19—H19C	0.9600
C4—C9	1.386 (3)	C20—C21	1.374 (4)
C5—C6	1.394 (3)	C20—H20	0.9300
C5—H5	0.9300	C21—H21	0.9300
C6—C7	1.384 (3)		
			
O1W—Zn1—O2W	117.76 (7)	C7—C8—C2	120.5 (2)
O1W—Zn1—O3	99.49 (7)	C4—C9—C8	121.6 (2)
O2W—Zn1—O3	94.51 (7)	C4—C9—H9	119.2
O1W—Zn1—N1	115.84 (7)	C8—C9—H9	119.2
O2W—Zn1—N1	124.77 (8)	N1—C10—C11	123.3 (2)
O3—Zn1—N1	89.06 (7)	N1—C10—H10	118.3
O1W—Zn1—N2	93.17 (7)	C11—C10—H10	118.3
O2W—Zn1—N2	89.03 (7)	C12—C11—C10	119.9 (2)
O3—Zn1—N2	163.37 (7)	C12—C11—H11	120.1
N1—Zn1—N2	75.66 (7)	C10—C11—H11	120.1
C2—O3—Zn1	133.53 (15)	C11—C12—C14	117.2 (2)
C3—O5—H5A	109.5	C11—C12—C13	121.7 (2)
Zn1—O1W—H1WA	110.1	C14—C12—C13	121.0 (2)
Zn1—O1W—H1WB	111.0	C12—C13—H13A	109.5
H1WA—O1W—H1WB	112.2	C12—C13—H13B	109.5
Zn1—O2W—H2WA	119.9	H13A—C13—H13B	109.5
Zn1—O2W—H2WB	120.2	C12—C13—H13C	109.5
H2WA—O2W—H2WB	109.6	H13A—C13—H13C	109.5
C10—N1—C15	117.7 (2)	H13B—C13—H13C	109.5
C10—N1—Zn1	124.60 (16)	C15—C14—C12	120.5 (2)
C15—N1—Zn1	117.70 (14)	C15—C14—H14	119.7
C21—N2—C16	118.1 (2)	C12—C14—H14	119.7
C21—N2—Zn1	125.98 (17)	N1—C15—C14	121.4 (2)
C16—N2—Zn1	115.76 (15)	N1—C15—C16	115.7 (2)
O1—C1—O2	125.7 (2)	C14—C15—C16	123.0 (2)
O1—C1—C4	117.5 (2)	N2—C16—C17	121.5 (2)
O2—C1—C4	116.8 (2)	N2—C16—C15	114.79 (19)
O4—C2—O3	126.4 (2)	C17—C16—C15	123.7 (2)
O4—C2—C8	118.0 (2)	C16—C17—C18	120.8 (2)
O3—C2—C8	115.6 (2)	C16—C17—H17	119.6
O6—C3—O5	123.0 (2)	C18—C17—H17	119.6
O6—C3—C6	119.6 (2)	C17—C18—C20	116.4 (2)
O5—C3—C6	117.45 (19)	C17—C18—C19	121.4 (2)
C5—C4—C9	119.4 (2)	C20—C18—C19	122.2 (2)
C5—C4—C1	121.50 (19)	C18—C19—H19A	109.5
C9—C4—C1	119.1 (2)	C18—C19—H19B	109.5
C4—C5—C6	119.9 (2)	H19A—C19—H19B	109.5
C4—C5—H5	120.0	C18—C19—H19C	109.5
C6—C5—H5	120.0	H19A—C19—H19C	109.5
C7—C6—C5	120.0 (2)	H19B—C19—H19C	109.5
C7—C6—C3	120.7 (2)	C21—C20—C18	120.3 (2)
C5—C6—C3	119.27 (19)	C21—C20—H20	119.8
C6—C7—C8	120.7 (2)	C18—C20—H20	119.8
C6—C7—H7	119.6	N2—C21—C20	122.7 (2)
C8—C7—H7	119.6	N2—C21—H21	118.6
C9—C8—C7	118.4 (2)	C20—C21—H21	118.6
C9—C8—C2	121.1 (2)		
			
O1W—Zn1—O3—C2	57.8 (2)	O4—C2—C8—C9	−173.5 (2)
O2W—Zn1—O3—C2	−61.4 (2)	O3—C2—C8—C9	5.7 (3)
N1—Zn1—O3—C2	173.8 (2)	O4—C2—C8—C7	5.3 (3)
N2—Zn1—O3—C2	−163.2 (2)	O3—C2—C8—C7	−175.5 (2)
O1W—Zn1—N1—C10	89.3 (2)	C5—C4—C9—C8	0.6 (3)
O2W—Zn1—N1—C10	−105.6 (2)	C1—C4—C9—C8	179.8 (2)
O3—Zn1—N1—C10	−10.8 (2)	C7—C8—C9—C4	−0.3 (3)
N2—Zn1—N1—C10	175.8 (2)	C2—C8—C9—C4	178.6 (2)
O1W—Zn1—N1—C15	−88.84 (17)	C15—N1—C10—C11	−0.6 (4)
O2W—Zn1—N1—C15	76.23 (18)	Zn1—N1—C10—C11	−178.77 (19)
O3—Zn1—N1—C15	171.07 (17)	N1—C10—C11—C12	0.7 (4)
N2—Zn1—N1—C15	−2.30 (16)	C10—C11—C12—C14	−0.6 (4)
O1W—Zn1—N2—C21	−63.8 (2)	C10—C11—C12—C13	179.0 (2)
O2W—Zn1—N2—C21	54.0 (2)	C11—C12—C14—C15	0.5 (4)
O3—Zn1—N2—C21	156.6 (2)	C13—C12—C14—C15	−179.0 (2)
N1—Zn1—N2—C21	−179.6 (2)	C10—N1—C15—C14	0.5 (3)
O1W—Zn1—N2—C16	121.32 (17)	Zn1—N1—C15—C14	178.81 (18)
O2W—Zn1—N2—C16	−120.94 (18)	C10—N1—C15—C16	−179.1 (2)
O3—Zn1—N2—C16	−18.3 (4)	Zn1—N1—C15—C16	−0.8 (3)
N1—Zn1—N2—C16	5.44 (17)	C12—C14—C15—N1	−0.5 (4)
Zn1—O3—C2—O4	3.8 (4)	C12—C14—C15—C16	179.1 (2)
Zn1—O3—C2—C8	−175.34 (15)	C21—N2—C16—C17	−3.1 (4)
O1—C1—C4—C5	−175.4 (2)	Zn1—N2—C16—C17	172.22 (19)
O2—C1—C4—C5	3.9 (3)	C21—N2—C16—C15	177.2 (2)
O1—C1—C4—C9	5.5 (3)	Zn1—N2—C16—C15	−7.5 (3)
O2—C1—C4—C9	−175.3 (2)	N1—C15—C16—N2	5.6 (3)
C9—C4—C5—C6	−0.3 (3)	C14—C15—C16—N2	−174.0 (2)
C1—C4—C5—C6	−179.5 (2)	N1—C15—C16—C17	−174.1 (2)
C4—C5—C6—C7	−0.3 (3)	C14—C15—C16—C17	6.2 (4)
C4—C5—C6—C3	−179.9 (2)	N2—C16—C17—C18	2.2 (4)
O6—C3—C6—C7	178.6 (2)	C15—C16—C17—C18	−178.1 (2)
O5—C3—C6—C7	−1.8 (3)	C16—C17—C18—C20	0.2 (4)
O6—C3—C6—C5	−1.8 (3)	C16—C17—C18—C19	−179.6 (3)
O5—C3—C6—C5	177.8 (2)	C17—C18—C20—C21	−1.6 (4)
C5—C6—C7—C8	0.7 (3)	C19—C18—C20—C21	178.2 (3)
C3—C6—C7—C8	−179.8 (2)	C16—N2—C21—C20	1.7 (4)
C6—C7—C8—C9	−0.3 (3)	Zn1—N2—C21—C20	−173.1 (2)
C6—C7—C8—C2	−179.2 (2)	C18—C20—C21—N2	0.7 (4)

### Hydrogen-bond geometry (Å, °)

**Table:**

*D*—H···*A*	*D*—H	H···*A*	*D*···*A*	*D*—H···*A*
O5—H5A···O6^i^	0.82	1.79	2.603 (2)	171
O1W—H1WA···O1^ii^	0.84	1.81	2.635 (2)	165
O1W—H1WB···O2^iii^	0.84	1.75	2.593 (2)	171
O2W—H2WA···O2^iv^	0.85	1.86	2.688 (2)	166
O2W—H2WB···O4^v^	0.84	1.79	2.633 (2)	176

Symmetry codes: (i) −*x*−1, −*y*+1, −*z*+2; (ii) −*x*+1, −*y*+1, −*z*+1; (iii) *x*, *y*+1, *z*; (iv) −*x*, −*y*+1, −*z*+1; (v) −*x*, −*y*+2, −*z*+1.

## References

[bb1] Brandenburg, K. (1999). *DIAMOND* Crystal Impact GbR, Bonn, Germany.

[bb2] Bruker (2001). *SADABS* Bruker AXS Inc., Madison, Wisconsin, USA.

[bb3] Bruker (2007). *APEX2* and *SAINT* Bruker AXS Inc., Madison, Wisconsin, USA.

[bb4] Desiraju, G. R. (2004). *Hydrogen Bonding* in *Encyclopedia of Supramolecular Chemistry*, edited by J. L. Atwood & J. W. Steed, pp. 658–665. New York: Marcel Dekker Inc.

[bb5] Maspoch, D., Ruiz-Molina, D. & Veciana, J. (2007). *Chem. Soc. Rev.* **36**, 770–818. 10.1039/b501600m 17471401

[bb6] Ockwig, N. W., Delgado-Friedrichs, O., O’Keefee, M. & Yaghi, O. M. (2005). *Acc. Chem. Res.* **38**, 176–182. 10.1021/ar020022l 15766236

[bb7] Sheldrick, G. M. (2008). *Acta Cryst.* A**64**, 112–122. 10.1107/S0108767307043930 18156677

[bb8] Zang, S.-Q., Liang, R., Fan, Y.-J., Hou, H.-W. & Mak, T. C. W. (2010). *Dalton Trans.* pp. 8022–8032. 10.1039/c0dt00374c 20657949

[bb9] Zang, S.-Q., Su, Y., Li, Y.-Z., Ni, Z.-P. & Meng, Q.-J. (2006). *Inorg. Chem.* **45**, 174–180. 10.1021/ic051502m 16390053

